# Relating Functional Connectivity and Alcohol Use Disorder: A Systematic Review and Derivation of Relevance Maps for Regions and Connections

**DOI:** 10.1002/hbm.70156

**Published:** 2025-02-07

**Authors:** Marco Bottino, Natálie Bocková, Nico W. Poller, Michael N. Smolka, Justin Böhmer, Henrik Walter, Michael Marxen

**Affiliations:** ^1^ Department of Psychiatry and Psychotherapy Technische Universität Dresden Dresden Germany; ^2^ Department of Psychiatry and Psychotherapy CCM Charité – Universitätsmedizin Berlin, Corporate Member of Freie Universität Berlin, Humboldt‐Universität Zu Berlin, and Berlin Institute of Health Berlin Germany; ^3^ Institute of Medical Psychology Charité – Universitätsmedizin Berlin, Corporate Member of Freie Universität Berlin, Humboldt‐Universität Zu Berlin, and Berlin Institute of Health Berlin Germany

**Keywords:** addiction, alcohol use disorder, functional magnetic resonance imaging, meta‐analysis, relevance maps, resting‐state functional connectivity

## Abstract

Alcohol Use Disorder (AUD), a prevalent and potentially severe psychiatric condition, is one of the leading causes of morbidity and mortality. This systematic review investigates the relationship between AUD and resting‐state functional connectivity (rsFC) derived from functional magnetic resonance imaging data. Following the PRISMA guidelines, a comprehensive search yielded 248 papers, and a screening process identified 39 studies with 73 relevant analyses. Using the automated anatomical labeling atlas for whole‐brain parcellation, relevance maps were generated to quantify associations between brain regions and their connections with AUD. These outcomes are based on the frequency with which significant findings are reported in the literature, to deal with the challenge of methodological diversity between analyses, including sample sizes, types of independent rsFC features, and AUD measures. The analysis focuses on whole‐brain studies to mitigate selection biases associated with seed‐based approaches. The most frequently reported regions include the middle and superior frontal gyri, the anterior cingulate cortex, and the insula. The generated relevance maps can serve as a valuable tool for formulating hypotheses and advancing our understanding of AUD's neural correlates in the future. This work also provides a template on how to quantitatively summarize a diverse literature, which could be applied to more specific aspects of AUD, including craving, relapse, binge drinking, or other diseases.

## Graphical abstract

Investigating the interplay between brain connectivity and alcohol use disorder (AUD), this review synthesizes findings and methodologies from resting‐state fMRI studies to develop relevance maps that link brain regions and functional connectivity to disorder manifestations, serving as a foundational resource for future hypotheses and methodologies in AUD research.

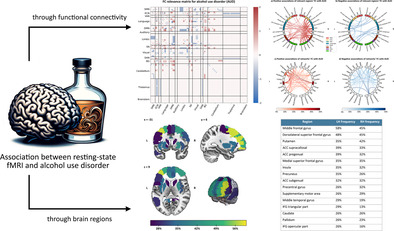

## 1 Introduction

### 1.1 Alcohol Use Disorder (AUD) and Its Global Impact

Alcohol Use Disorder (AUD) is a condition characterized by a problematic pattern of alcohol use that leads to significant distress and social, occupational, or health problems. Across the world, AUD has emerged as one of the most prevalent and severe psychiatric conditions, affecting millions of individuals and contributing significantly to the global burden of disease (Shield et al. 2020). Alcohol consumption is the seventh largest risk factor for death (Griswold et al. 2018). Approximately 283 million adults worldwide are estimated to suffer from AUD (Glantz et al. 2020), which stands as a global public health challenge with profound implications for individuals and societies. The conceptualization of AUD has evolved over time across different editions of the Diagnostic and Statistical Manual of Mental Disorders (DSM). In its fourth edition (DSM‐IV; American Psychiatric Association 1994), and its text revision (DSM‐IV‐TR; American Psychiatric Association 2000), the manual differentiated between “alcohol abuse” and “alcohol dependence,” delineating distinct criteria for each diagnosis. However, the subsequent edition (DSM‐5; American Psychiatric Association 2013) merged these previously separate categories into a singular diagnosis of AUD, now viewed as a spectrum ranging from mild to severe based on the number of diagnostic criteria fulfilled. The research findings discussed in this review primarily address the condition of alcohol dependence as outlined in the DSM‐IV‐TR and AUD as characterized in the DSM‐5. This text will adopt the term AUD to refer to these conditions.

### 1.2 Brain Imaging as a Tool for Investigating AUD

Functional magnetic resonance imaging (fMRI) is a brain imaging technique based on the blood‐oxygen‐level‐dependent (BOLD) signal, which has been frequently used to characterize the neural substrates of mental disorders of different types, including substance use disorders (Abraham et al. 2017; Geng et al. 2017; Lord et al. 2012; Makris et al. 2006; Orr et al. 2013; Roberts et al. 2017; Yuan et al. 2016). It is classically divided into task‐based and resting‐state fMRI. In the first case, a subject's reaction to specific tasks or cues produces alterations in the signal, which can be used to highlight task‐specific activity in selected brain regions. On the other hand, resting‐state functional connectivity (rsFC) exploits low‐frequency fluctuations of the signal to find common temporal patterns of activation between regions (functional connections), in other words, the inter‐regional coupling of intrinsic neuronal activity while the subject is awake and at rest (Biswal et al. 1995). Sets of brain regions with similar signal time courses are called functional networks.

The relationship between AUD and changes in brain structure and function has been extensively explored in the literature, with reviews focusing on how various imaging modalities assist in identifying the effects of the disorder, for example, Dupuy and Chanraud (2016), Fritz, Klawonn, and Zahr (2022), Gowin et al. (2023), and Nutt et al. (2021). Concerning brain anatomy, there is a consensus in these reviews on an association between AUD and brain atrophy in some key regions, like anterior cingulate cortex (ACC), dorsolateral prefrontal cortex (PFC), insula, amygdala, hippocampus, thalamus, cerebellum and striatal regions like caudate, putamen and nucleus accumbens (Nacc) (Li et al. 2021; Yang et al. 2016). Furthermore, fMRI studies helped shed light on the role of these regions in neurocognitive dysfunctions linked to the disorder such as altered alcohol cue‐reactivity (Schacht, Anton, and Myrick 2013; Zeng et al. 2021), reward processing (Galandra et al. 2018; Zeng et al. 2023), or emotional regulation (Stellern et al. 2023; Weiss et al. 2022). In this regard, task‐based studies identified that, in AUD, visual alcohol cues elicit a pronounced hemodynamic response in key areas involved in the reward circuitry, such as the dopaminergic mesolimbic pathway, which comprises the ventral tegmental area (VTA) and extends through the amygdala, Nacc, ACC, insula, and prefrontal regions (Schacht, Anton, and Myrick 2013; Zeng et al. 2021). This response is indicative of altered reward processing, where individuals with AUD show heightened reactivity to alcohol‐related cues and diminished response to non‐drug stimuli, pointing to a devaluation of natural rewards (Wrase et al. 2007). Similarly, emotional regulation is significantly affected, with a marked decrease in the ability to process and respond to emotional signals (Wilcox, Pommy, and Adinoff 2016), especially fear (O'Daly et al. 2012). This is associated with reduced activity in the orbitofrontal cortex (OFC), insula, and amygdala, and an increased reliance on prefrontal regions over temporal limbic areas during emotionally demanding tasks, indicating compromised emotional control (Donadon and Osório 2014).

### 1.3 Resting‐State Functional Connectivity and AUD

Concerning rsFC, the literature highlights a disruption in neural coherence within and between brain networks in individuals with AUD, including decreased within‐network connectivity and aberrant expansion to nonspecialized regions (Müller‐Oehring et al. 2015). This dedifferentiation reflects a broader impairment in executive control and cognitive performance, particularly within the default mode network (DMN), salience network (SN), and executive control network (ECN) (Fede et al. 2019; Zhu, Cortes, et al. 2015). As a consequence of these effects, Dupuy and Chanraud (2016) define AUD as a “disconnection syndrome.” These reviews provide a general overview of the association between AUD and the changes in brain structure and function but, to our knowledge, no review specifically focused on the relationship between rsFC and AUD yet. Other reviews explored the relationship of rsFC pathways with addiction in general (Pariyadath, Gowin, and Stein 2016; Sutherland et al. 2012; Tolomeo and Yu 2022). Their findings, despite not specific to AUD, highlighted again the involvement of DMN and ECN and the driving role of the salience network (SN), particularly of the insula and dorsal anterior cingulate cortex (dACC).

### 1.4 Purpose and Structure of the Review

In this systematic review, we will synthesize studies that investigated rsFC in AUD and discuss the most common analytical approaches, hypotheses, and results. The diverse methodologies used in the field preclude conventional meta‐analyses; however, we will discuss this heterogeneity in depth throughout the review. Furthermore, we will summarize the findings in terms of relevance maps. A relevance map is a brain map indicating in a semi‐quantitative way how often a brain region or connection has been associated with a clinically relevant AUD parameter in the included literature, that is, they indicate how relevant a brain region or a connection is for AUD according to the current state of art. Therefore, it is not based on theory but on the empirical evidence available. Our relevance maps are based on a particular brain parcellation, namely the automated anatomical labeling atlas, version 3.1 (AAL3) (Rolls et al. 2020), and a specific algorithm described in the Methods section to translate all reported findings into this space. The purpose of the relevance maps is to provide a quantitative synthesis of the literature, offering a structured framework to support hypothesis generation and guide future studies. The purpose of the relevance maps is to provide a quantitative synthesis of the literature, offering a structured framework to support hypothesis generation and guide future studies. We decided to produce two outcomes: an FC relevance matrix that indicates the positive or negative association of any particular connection with AUD, and a ranking of the regions according to the percentage of analyses that identified it as significantly associated with AUD. To address the methodological variability across studies, we distinguish between whole‐brain analyses and seed‐based analyses. Whole‐brain analyses consider data from the entire brain or predefined parcellations that cover at least all of the cortex without a priori selection of specific regions, while seed‐based analyses focus only on a subset of regions chosen beforehand, typically to increase statistical power or test specific hypotheses. We chose to use only whole‐brain analyses in the generation of the relevance maps in the first place to reduce selection bias. However, we also performed a supplementary comparison with relevance maps generated from seed‐based analyses to evaluate potential biases and identify regions that may be over‐ or underrepresented when selected as seeds. In the following sections, we will first describe the process of paper selection, which followed the Preferred Reporting Items for Systematic Reviews and Meta‐Analyses (PRISMA) guidelines (Page et al. 2021). Then, our method of extracting relevant information from the included studies to create the relevance maps will be explained. The results will provide an overview of the information extracted from the papers and the relevance maps' outcome, as well as a comparison between the outcomes of whole‐brain and seed‐based analyses. Finally, we will discuss the results, potential biases, limitations, and avenues for further development of the relevance map approach.

## 2 Methods

### 2.1 Search Strategy and Paper Selection

**FIGURE 1:**
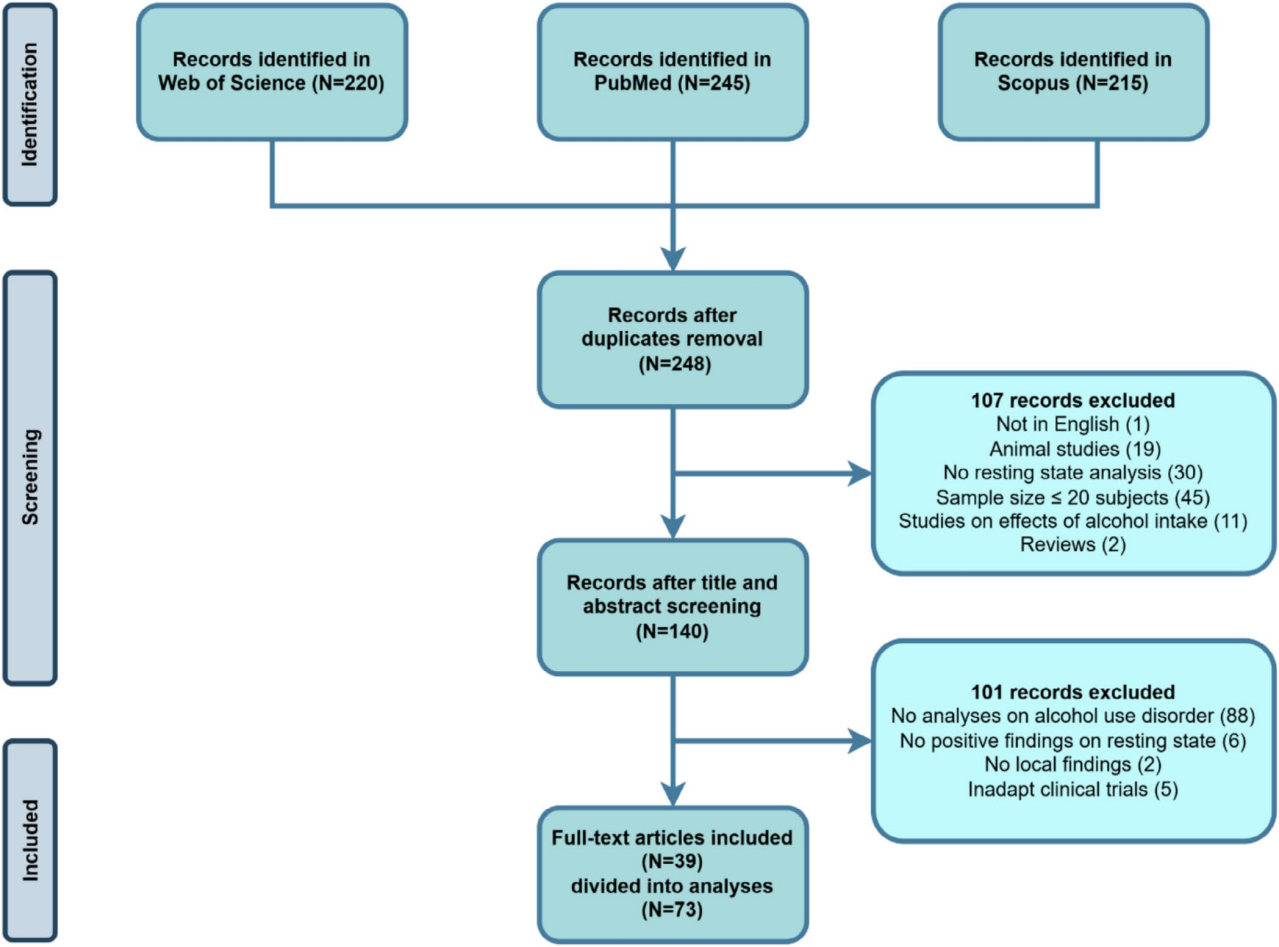
Paper selection flowchart according to PRISMA. The process includes the search on three different databases, removing duplicates, screening titles and abstracts, and a final control for eligibility on the full‐text level, resulting in 39 included papers with 73 distinct analyses linking rsFC and alcohol use disorder.

On April 10th, 2023, we searched for relevant literature on the topic in three databases: PubMed (https://pubmed.ncbi.nlm.nih.gov/), Scopus (https://www.scopus.com/), and Web of Science (https://www.webofscience.com/wos). We used the following query: “alcohol” AND “connectivity” AND (“resting” OR “rest”), and the type of document was limited to the article format. No limits on the date of publication were used. The search led to 248 papers, which were divided among three reviewers (MB, NB, and NWP) for screening. The screening (see Figure 1) consisted of verifying from the title and abstract of the record whether the paper met any of the exclusion criteria. We excluded studies based on animals, studies without resting‐state fMRI (rs‐fMRI) analysis, studies with 20 subjects or less in the AUD group (or in total when no group difference was present), studies not focusing on AUD, and studies on acute alcohol effects. In ambiguous cases where retrieving this information from the title or abstract was impossible, the full text would be consulted for clarification. In the end, 39 records were included, the main characteristics of which are summarized in Table 1.

**TABLE 1:** Summary of included studies relating rsFC to AUD.

References	Paper index	Whole brain?	Sample size	Independent variable	Type of analysis	Main results linking resting‐state fMRI to alcohol use disorder
Bordier et al. (2022)	1	Yes	*N* = 69 (AUD: 35, HC: 34)	Participation coefficient	Group comparison of participation coefficient, AUD‐HC	Increased centrality of the anterior insula in AUD patients predicted by regression
Bracht et al. (2021)	2	No	*N* = 57 (AUD: 39, HC: 18)	Seed‐Seed FC of Nacc‐OFC	Correlation with OCDS craving score	Nacc‐OFC FC pos. corr. with craving in AUD
Camchong et al. (2022)	3	No	*N* = 45 (ABS: 25, REL: 20)	Within‐network FC in predefined networks	Correlation with OCDS craving score	Lower connectivity in relapsers than abstainers in all the networks at baseline
					Correlation with time to relapse (days)	Decreased connectivity in the networks predicts the time to relapse
Canessa et al. (2022)	4	Yes	*N* = 40 (AUD: 22, HC: 18)	Level of coherence in intra‐network activity, frequency measures	Group comparison of coherent activity, AUD‐HC	Decreased low (≤ 0.1 Hz) and very low (< 0.05 Hz) frequency power and increased high (> 0.1 Hz) frequency power in sensorimotor, visual, basal ganglia, and executive networks
Dean et al. (2020)	5	No	*N* = 158	Seed‐voxel FC of amygdala	AUDIT score	Pos. corr. between R amygdala‐R temporal fusiform gyrus connectivity and AUDIT
Deng et al. (2022)	6	No		Seed‐voxel FC of precentral, postcentral, and R fusiform gyri	Comparison between AUD and HC	Decreased FC of seeds with R lingual, insula, L middle cingulum, and L superior temporal cortex
		No		Seed‐voxel FC of precentral, postcentral, and R fusiform gyri	Comparison between REL and ABS	Decreased FC between L precentral and L cerebellum
		No		Previous significant FC	Multivariate logistic regression of yes versus no alcohol dependence	Some of the previous significant results were associated with alcohol dependence from the model
Duan et al. (2022)	7	Yes	*N* = 90 (AUD‐NCI: 50, ARCI: 29, HC: 11)	Global FC density (FCD)	Comparison between groups (AUD‐NCI vs. HC)	Decreased global FCD between AUD noncognitive impairment and HC in MFG, calcarine, and L IOL
				Local FCD	Comparison between groups (AUD‐NCI vs. HC)	Decreased local FCD between AUD noncognitive impairment and HC in ParaHipp and R lingual
				Long‐range FCD	Comparison between groups (AUD‐NCI vs. HC and AUD‐NCI vs. ARCI)	Decreased long‐range FCD in MFG and VMPFC
				Global FCD	Correlation with ADS score	Pos. corr. between global FCD and ADS in L IOL
Farokhnia et al. (2022)	8	Yes	*N* = 181	Normalized within‐network connectivity	2 × 2 study high/low AUDIT score, risk allele yes or no comparison	In the group with rs6923761 risk allele, greater connectivity in high AUDIT in aSN and visual net.
						In the group with rs1042044 risk allele, greater connectivity in high AUDIT in DMN and BG network
Fede et al. (2019)	9	Yes	*N* = 73 (primary: 49, validation: 24)	Resting neural engagement within‐networks	MANCOVA model of AUDIT, whole sample and separated for age	Within‐network connectivity correlates with AUDIT
				Within‐ and between‐network FC	Random forest regression of AUDIT with multimodal input	Resting state within‐ and between‐net. connectivity best predicts AUDIT, especially BG connectivity
Fein et al. (2017)	10	No	*N* = 109 (HC: 69, LTAA: 40)	Seed‐voxel RSS of Nacc and subgenual ACC	Group comparison between LTAA and HC	LTAA had higher RSS than HC in ECN and emotional regulation net. and lower in appetitive drive net.
Galandra et al. (2019)	11	No	*N* = 40 (AUD: 22, HC: 18)	Seed‐Seed FC of BG, Thalamus, ECN, aSN	Group comparison, AUD‐HC	Decreased FC between thalamus and BG with ECN and aSN
					Correlation with average daily alcohol dose	Negative correlation of BG‐ECN between‐net. connectivity with daily alcohol consumption
Gazula et al. (2023)	12	Yes	cVEDA: *N* = 1979 (risk 0: 1312, risk 1: 559, risk 2: 108) IMAGEN: *N* = 1979 (risk 0: 1312, risk 1: 559, risk 2: 108)	Static functional network connectivity	Group comparison, alcohol risk 0 (low)–alcohol risk 1 (moderate)–alcohol risk 2 (high)	Increased conn. mostly linked to DMN and, to a lesser extent, cognitive control and subcortical
Gerchen et al. (2019)	13	No	*N* = 106 (AUD: 66, HC: 40)	Gradient of conn. between frontal regions and striatum (PeaCoG)	Group comparison, AUD‐HC	Significantly lower values in the AUD group (greater conn. in ventral part of the striatum)
					Corr. with AASE abstinence score	Neg. corr. between PeaCoG and AASE score
					Correlation with ADS score	Pos. corr. between PeaCoG and ADS score
					Correlation with AUQ score	Neg. corr. between PeaCoG and AUQ score
Guo et al. (2019)	14	Yes	*N* = 89 (HC: 35, AUD: 24, AUD + nicotine: 30)	Voxel‐mirrored homotopic connectivity‐frequency measure	Group comparison, AUD‐HC	Decreased homotopic FC in CPL, increased in MFG
		No		Homotopic FC in significant ROIs from previous results	Corr. of significant seeds with clinical variables in typical and slow‐4 bands	Neg. corr. of homotopic CPL FC with alcohol intake and pos. corr. of homotopic MFG with AUDIT score
Honnorat et al. (2022)	15	Yes	*N* = 292 (AUD: 107, AUD + HIV: 38, HIV: 47, HC: 100)	Between‐net. FC	Group comparison, AUD‐HC	Decreased conn. ACC‐OFC, OFC‐SM area, ACC‐hippocampal regions, increased conn. SFG‐piCPL
		No		Between‐net. FC of significant connections	Correlation with total lifetime alcohol consumption	Negative correlation between alcohol consumption and ACC‐OFC connection
Jansen et al. (2015)	16	No	*N* = 75 (AUD: 38, HC: 37)	Within‐net. conn. (dual regression) of FPn and reward net.	Group comparison of within‐network FC, AUD‐HC	AUD showed greater conn. in left FPn and reward/motivation network
		No		Between‐network FC of FPn and reward net.	Group comparison or between‐network FC, AUD‐HC	AUD showed a stronger conn. between L and R FP networks, neg. corr. With AUDIT score
Kamarajan et al. (2020)	17	No	*N* = 60 (AUD: 30, HC: 30)	rs‐fMRI FC of DMN, task‐fMRI, impulsivity scores	AUD vs. HC classification (Random Forest)	Classification accuracy of 76.67%, with importance measure and effect direction for significant inputs
Kamarajan et al. (2022)	18	No	*N* = 60 (AUD: 30, HC: 30)	rs‐fMRI FC of reward net., task‐fMRI, impulsivity scores	AUD vs. HC classification (Random Forest)	Classification accuracy of 86.67%, with importance measure and effect direction for significant inputs
Kim et al. (2017)	19	No	*N* = 54 (AUD: 26, HC: 28)	Seed‐seed of ROIs from DMN, DAN, ECN, SN, SM network	Group comparison of within‐network FC, AUD‐HC	Decreased AUD conn. In ECN and between the medial temporal complex and IPS
				Group, AUD‐HC	Multivariate hierarchical linear regression, group as covariate	COTC and DAN within‐network avg. connectivity predicted significantly by group
Kohno et al. (2017)	20	No	*N* = 69 (HC: 26, REL: 27, ABS: 16)	Seed‐voxel FC of the whole striatum	Group comparison, AUD‐HC, and REL‐ABS	Differences in FC in both directions between AUD‐HC and REL‐ABS
				FC between significant ROIs and striatum	Correlation of FC with VAS craving score	Group interaction in corr. striatum‐posterior insula, striatum‐R dlPFC and ECN‐amygdala
		No		FC of ECN and reward/salience network	Group comparison, REL‐ABS	Greater conn. In REL than ABS between ECN and R amygdala and reward/salience net. with putamen, insula and precuneus
Le et al. (2021)	21	No	*N* = 61	Seed‐voxel FC of the hypothalamus and ventral striatum	Correlation with AUDIT Score	Pos. corr. of hypothalamus‐hipp and neg. corr. Of MFG‐ventral striatum FC with AUDIT score
Lesnewich et al. (2022)	22	No	*N* = 42 (AUD: 22, HC: 20)	FC between ECN core nodes, AUD status	Poisson GLM of past month drinking episodes, cross‐sectional	The cross‐sectional model yielded five significant associations between CEN connectivity and BD incidence
					Poisson GLM of past month's drinking episodes, perspective	Stronger FP conn. between the R dlPFC and L PPC predicted greater increases in BD incidence over time
Maleki et al. (2022)	23	Yes	*N* = 55 (AUD: 23, HC: 32)	Within‐network connectivity (dual regression)	Group comparison, AUD‐HC	Differences in FC in both directions
				Connectivity strength between components	Correlation with AUD‐related measures	Pos. corr. of R putamen‐R SMA and R putamen‐L precuneus with length of sobriety, neg. corr. of L precuneus‐MFG and L precuneus‐subcallosal cortex with the number of daily drinks
Manuweera et al. (2022)	24	No	*N* = 150 (AUD: 75, HC: 75)	Seed‐voxel FC of anterior/posterior L/R insula	Group comparison, AUD‐HC	Significantly decreased connectivity for all seeds in AUD
Morris et al. (2016)	25	No	*N* = 134 (HC: 66, AUD: 36, BD: 32)	Seed‐voxel FC of the subthalamic nucleus	Group comparison between AUD, BD, and matching HC	Decreased conn. in BD and AUD of the subthalamic nucleus with IPC, Nacc, and subgenual ACC
				Seed‐voxel FC of the subthalamic nucleus	Correlation of FC with AUDIT score	Neg. corr. of subthalamic nucleus‐subgenual ACC FC with AUDIT score
Müller‐Oehring et al. (2015)	26	No	*N* = 53 (AUD: 27, HC: 26)	Seed‐voxel FC of PCC, SFG, ACC, SPL, postcentral, Heschl, calcarine	Group comparison in intensity, AUD‐HC	Differences in FC of the seeds selected between the groups
					Group comparison in FC cluster size, AUD‐HC	Expansion and restriction of different clusters of FC of the seeds selected
					Correlation with ACQ‐R score	Negative correlation of NACC‐L amygdala FC with ACQ‐R craving score
Müller‐Oehring et al. (2022)	27	Yes	*N* = 55 (AUD: 33, HC: 22)	FC matrix	Group comparison, AUD‐HC	Differences in both directions in FC between groups
					Correlation with lifetime alcohol consumption	Pos. corr. of cerebellar‐prefrontal FC with lifetime alcohol consumption
Song et al. (2021)	28	No	*N* = 58 (AUD: 28, HC: 30)	Within‐network FC	Group comparison, AUD‐HC	Decreased conn. in R SFG, R MFG, L IPS and R SFG
Suk, Hwang, and Cheong (2021)	29	Yes	*N* = 44 (AUD: 22, HC: 22)	ROI‐ROI functional connectivity	Group comparison, AUD‐HC	Increased conn. within DMN and SN and decreased conn. within ECN
Vergara et al. (2017)	30	Yes	*N* = 79 (AUD: 28, HC: 51)	FC matrix	Group comparison, AUD‐HC	Differences in FC across groups, isolating the results from AUD + SMK and SMK groups
		No		Significant FC from previous analysis	Regression of AUDIT score	Negative association of AUDIT score with postcentral‐fusiform and postcentral‐lingual gyri FC
Vergara, Espinoza, and Calhoun (2022)	31	Yes	*N* = 102 (AUD: 51, HC: 51)	FC matrix	Classification of AUD vs. HC with different machine learning approaches	Hypoconn. putamen‐frontal lobe and thalamus‐frontal lobe, and hyperconn. insula‐temporal gyrus were the most important features according to Random Forest importance
Wang, Zhao, et al. (2018)	32	Yes	*N* = 42 (AUD: 21, HC: 21)	Nodal efficiency of ROIs	Group comparison, AUD‐HC	AUD group exhibited alterations in brain network efficiency
				FC matrix	Group comparison, AUD‐HC	Decreased conn. between R DLPFC and R SOG
Wang, Fan, et al. (2018)	33	No	*N* = 87 (HC: 31, REL: 35, ABS: 21)	Seed‐voxel FC of cuneus, thalamus, R precuneus, R dlPFC, R dPCC	Group comparison, HC‐ABS‐REL	Increased neg. FC of R cuneus with regions from ECN and SN
				Seed‐voxel FC significant results from previous analysis	Logistic regression of relapse state using GMV and rsfMRI measures	R cuneus—R dlPFC associated positively with the prediction of relapse state at one‐month follow‐up
Weiland et al. (2014)	34	Yes	*N* = 470 (AUD: 383, HC: 87)	FC matrix	MANCOVA group comparison, AUD‐HC	Group differences in L ECN, sensorimotor, BG, and primary visual networks
		No		L ECN network connectivity strength	Correlation with AUDIT score	Neg. corr. with AUDIT between nodes of L ECN
Yang et al. (2021)	35	No	*N* = 134 (AUD: 68, HC: 68, REL: 35, ABS: 32)	Seed‐voxel FC of Nacc and medial PFC	Group comparison, AUD‐HC	Decreased AUD conn. in Nacc and mPFC with temporal, cingulate, postcentral, and fusiform gyri
					Group comparison, REL‐ABS	Decreased REL conn. of Nacc‐R ACC and mPFC‐L calcarine
					Partial correlation with relapse severity at follow‐up	mPFC‐R SFG FC has negative correlation with relapse severity
Zhu, Dutta, et al. (2015)	36	Yes	*N* = 140 (AUD: 68, HC: 72)	Within‐network connectivity (dual regression)	Group comparison, AUD‐HC	Greater within‐net. FC in AUD, correlated positively with ADS
Zhu, Cortes, et al. (2015)	37	Yes	*N* = 51 (AUD: 25, HC: 26)	Within‐network connectivity (dual regression)	Group comparison, AUD‐HC	Greater within‐net. FC in SN, DMN, OFC, L ECN, amygdala‐striatum networks
				Between‐network connectivity		Increased FC between L ECN, ASN, and SN
Zhu et al. (2018)	38	Yes	*N* = 92 (AUD: 46, HC: 46)	Within‐ and between‐network FC	Evaluation of FI in classification (AUD‐HC) according to feature selection	Within‐net. conn. of ECN and RN, as well as between conn. among ECN, RN, and DMN
Zhu et al. (2022)	39	Yes	*N* = 573 (Mild AUD: 58, Comorbid AUD: 46, Moderate AUD: 72, HC: 397)	FC matrix	Comparison between mild AUD and control in FC matrix	Significant differences in FC in frontal, parietal, subcortical, and default mode networks
					Comparison between comorbid AUD and control in FC matrix	
					Comparison between moderate AUD and control in FC matrix	

Abbreviations: AASE—alcohol abstinence self‐efficacy scale; ABS—abstainers; ACC—anterior cingulate cortex; ACQ‐R—alcohol craving questionnaire (short); ADS—alcohol dependence scale; AE—alcohol expectancy; AlcSI—alcohol substance involvement score; ant.—anterior; ARCI—alcohol‐related cognitive impairment; aSN—anterior salience network; AUD—alcohol use disorder; AUDIT—alcohol use disorder identification test; AUD‐NCI—alcohol use disorder—noncognitive impairment; AUQ—alcohol urge questionnaire; BD—binge drinkers; BG—basal ganglia; conn.—connectivity; COTC—cingulo opercular task control network; DAN—dorsal anterior network; diff.—difference; dlPFC—dorsolateral prefrontal cortex; DMN—default mode network; ECN—executive control network; FC—functional connectivity; FI—feature importance; FPn—fronto parietal network; HC—healthy controls; Hipp—hippocampus; ICA—independent component analysis; IFC—inferior frontal cortex; IOL—inferior occipital lobe; IPC—inferior parietal cortex; IPS—interparietal sulcus; L—left; LD—bight drinkers; LTAA—long term abstinent alcoholics; MFG—middle frontal gyrus; MFG—middle frontal gyrus; mPFC—medial prefrontal cortex; Nacc—nucleus accumbens; Neg. corr.—negative correlation; net.—network; OCDS—obsessive compulsive drinking scale; OFC—orbito frontal cortex; ParaHipp—Parahippocampus; PCC—posterior cingulate cortex; PeaCoG—peak connectivity locations on the gradient; piCPL—posterior inferior cerebellum; Pos. corr.—positive correlation; PPC—posterior parietal cortex; R—right; REL—relapsers; RSS—resting state synchrony; SFG—superior frontal gyrus; SM—sensorimotor; SN—salience network; SOG—superior occipital gyrus; SPL—superior parietal lobe; VAN—ventral attention network.

### 2.2 Creation of the Results Table

To synthesize the findings from the 39 included studies, the reviewers compiled essential information into a structured table. Each row in this table represents a unique significant finding from a study, while the columns capture specific features of interest, including information on sample size, participant demographics (gender and age), the type of input used for the model, the variable predicted or compared among groups, and the size, significance, and direction of the effect, when available. Given the substantial disparities regarding this information across studies, we decided not to consider these features when defining the algorithm for the relevance map creation, however, we still investigated how the papers vary in the most relevant methods and datasets used. A more detailed description of the main features investigated is provided in the next section. The review process was conducted independently by each reviewer, with MB conducting a comprehensive review of the compiled data for accuracy and consistency after all papers were analyzed in detail. Each paper could present one or more analyses, which were considered separately in the data collection process resulting in a total of 73 different analyses.

The papers varied widely in their analytical approaches, particularly in whether they examined the entire brain or focused on specific regions of interest, and in the type of FC results reported—either between two regions or within a single region. For each result, the relevant information used to create the relevance maps would be the connection or region associated with the disorder and the effect direction. To address the variability in terminology for brain regions and networks across these studies, we adopted the AAL3 as our reference parcellation (Rolls et al. 2020). We chose this atlas for the clear correspondence of its regions with known anatomical regions, the detailed cortical and subcortical subdivision, the hemispheric division, the reasonable specificity, and its popularity in the literature. Although AAL3 does not categorize regions into functional networks, we grouped the atlas regions into principal brain networks based on the criteria outlined by Briggs et al. (2018). Table 2 lists all atlas regions and their corresponding functional network.

**TABLE 2:** List of regions of automated anatomical labelling atlas, version 3.1, divided according to functional networks.

Network	Regions
Auditory network	Rolandic operculum and Heschl's gyrus
Brainstem	Ventral tegmental area (VTA), substantia nigra—pars compacta, substantia nigra—pars reticulata, red nucleus, locus coeruleus, and Raphe nucleus
Basal ganglia (BG)	Caudate, putamen, pallidum, and nucleus accumbens (Nacc)
Executive control network (ECN)	Dorsolateral superior frontal gyrus (SFG), middle frontal gyrus (MFG), and lateral orbital gyrus
Cerebellum	Crus I and II of cerebellar hemisphere, lobules I, II, III, IV‐V, VI, VIIB, VIII, IX, X of cerebellar hemisphere, lobules I‐II, III, IV‐V, VI, VII, VIII, IX, X of vermis
Default mode network (DMN)	Inferior frontal gyrus (IFG) pars orbitalis, superior medial orbital frontal gyrus, posterior cingulate cortex (PCC), precuneus, and anterior cingulate cortex subgenual and pregenual (ventral ACC)
Dorsal attention network (DAN)	Superior parietal gyrus (SPG) and inferior parietal gyrus (IPG)
Language network	IFG triangular part, medial SFG, angular gyrus, superior temporal gyrus (STG), middle temporal gyrus (MTG), inferior temporal gyrus (ITG)
Limbic/paralimbic network	Olfactory cortex, rectus, medial orbital gyrus (mOFC), anterior orbital gyrus (aOFC), posterior orbital gyrus (pOFC), hippocampus, amygdala, temporal pole, and superior and middle temporal gyrus
Salience network (SN)	Insula, middle cingulate cortex (MCC) and anterior cingulate cortex supracallosal (dorsal ACC)
Sensorimotor network	Precentral gyrus, postcentral gyrus, paracentral lobule, and supplementary motor area (SMA)
thalamus	Anteroventral, lateral posterior, ventral anterior, ventral lateral, ventral posterolateral, intralaminar, reuniens, mediodorsal medial magnocellular, mediodorsal lateral parvocellular, lateral geniculate, medial geniculate, pulvinar anterior, pulvinar medial, pulvinar lateral, and pulvinar inferior
Ventral attention network (VAN)	IFG opercular part and supramarginal gyrus
Visual network	Calcarine, cuneus, lingual, fusiform gyrus, superior occipital gyrus, middle occipital gyrus, and inferior occipital gyrus

*Note:* Aside from the lobules of the vermis and the Raphe nucleus, all regions are further divided into left and right according to hemisphere.

For each significant result, we created a new set of rows for all the corresponding regions in AAL3 according to the following criteria:

- If a set of MNI coordinates were reported (e.g., a peak value in a contrast map between AUD and a control group), the AAL3 region corresponding to those coordinates would be assigned.
- If a region was reported without a direct AAL3 match, we identified one or more regions best fitting (e.g., creating one row for each region within the left thalamus of AAL3 if the original analysis would report a result on “Thalamus L”).
- If a network was reported in the original analysis, we created one row for each AAL3 region belonging to this network, as described in Table 2. If a network did not belong to this subdivision, we defined the most fitting AAL3 regions according to the literature, starting from the paper citing it, and the first two criteria (e.g., for findings concerning within‐network connectivity of the emotional network, described for example as composed of bilateral amygdala, hippocampus, and Nacc, we created one row for each pairwise connection between the six corresponding AAL3 regions).

The complete results table can be found in Table S1.

### 2.3 Collection of Metadata About the Studies

To characterize the available studies, we will present histograms on the distribution of average age, gender, and general sample size. Furthermore, we will categorize all studies according to the following criteria:

- **Group division:** We looked at how subjects are grouped within each study, identifying whether there is a distinction between AUD subjects and healthy controls (HC), other subgroups, or no specific group division;
- **Diagnostic criteria used**: Given that all included studies involve AUD subjects, we examined the variety of diagnostic criteria used across these studies to identify AUD;
- **FC input used—**The processing of imaging data and definition of the input for the model can be done by focusing on specific seeds or the whole brain, but how individual ROIs are defined or the brain is divided into networks changes across the papers. We investigated the different approaches, such as atlas‐based parcellations, using independent component analysis (ICA) components to identify functional networks, or using masks or specific seed voxels derived from literature;
- **Seeds most commonly used—**In the context of seed‐based analyses, we will identify which regions and networks are most frequently chosen as seeds and by which studies;
- **Outcomes of interest—**Most analyses focus on finding statistical differences between predefined groups, but many different outcomes can be used to describe the severity of the disorder or some of its symptoms. We explored the most frequent scales used for severity, such as the Alcohol Use Disorders Identification Test (AUDIT) (Saunders et al. 1993) and the Alcohol Dependence Scale (ADS) (Skinner and Horn 1984), as well as other frequently measured aspects of the disorder;
- **Mediators investigated:** The effect of resting state changes on these scales can be studied directly through regression or correlation models or by investigating the role of possible mediators: we highlighted the most frequent ones and the papers using them.

For each category, we will only report elements that are featured in at least three different studies to ensure relevance and significance.

### 2.4 Creation of the Relevance Maps

**FIGURE 2:**
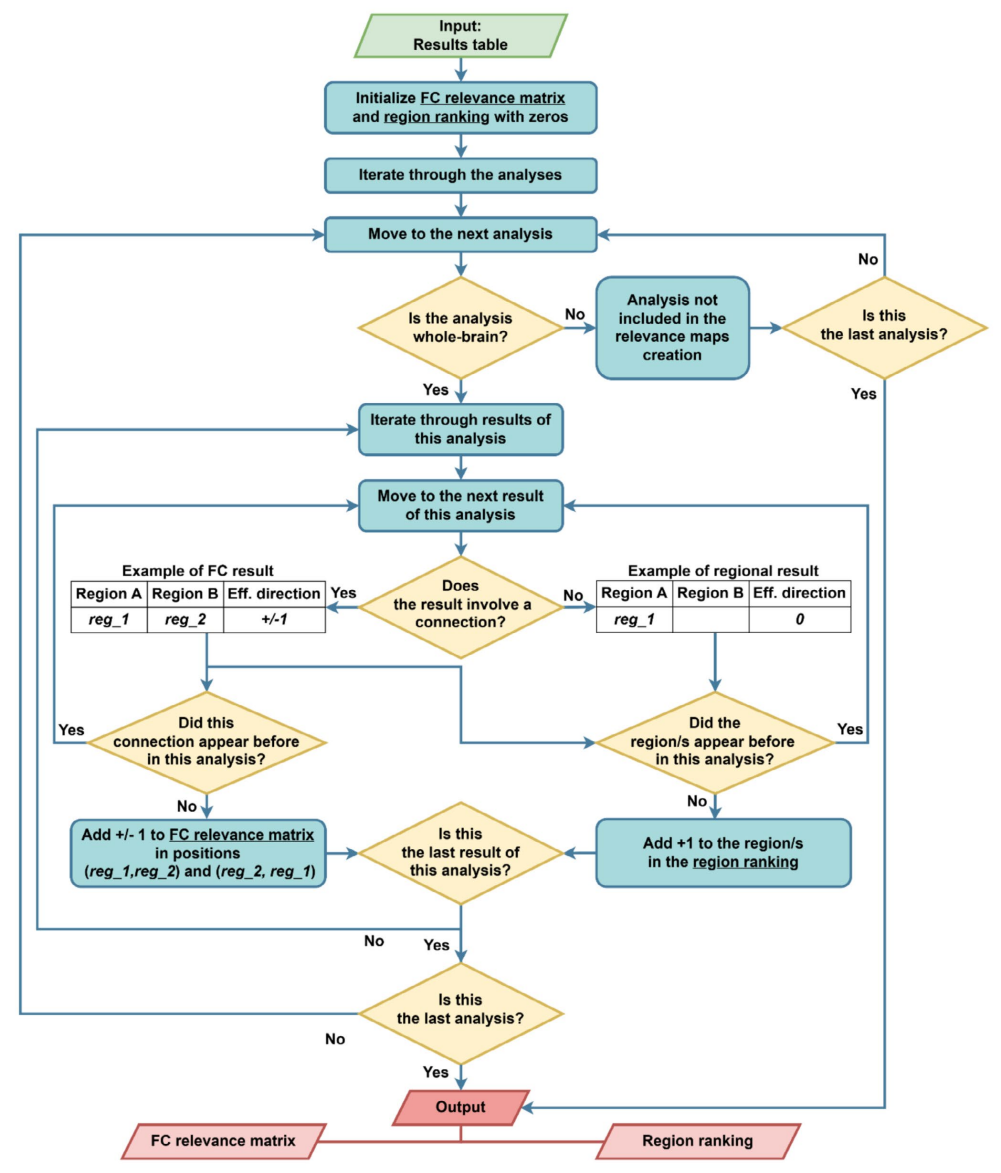
Flowchart explaining the process of the creation of the FC relevance matrix and the region ranking. The labels reg_1 and reg_2 represent two numeric IDs for regions of AAL3. AAL3—automated anatomical labelling atlas 3.1; FC—functional connectivity.

The main focus of this review is to summarize the significant results of the analyses in terms of (a) an FC relevance matrix for connections and (b) a ranking of the most relevant ROIs. The process used for their creation is shown in Figure 2. Our approach employs a nested iteration algorithm that first segments the results table based on the 73 analyses, then processes the data within each analysis‐specific sub‐table. To avoid the significant bias introduced by seed‐based analyses, our algorithm solely incorporates studies that examine the relationship between AUD and the entire brain, as determined by the first condition in the flowchart. The processes for generating the two types of relevance maps vary slightly, and we will detail them separately.

#### 2.4.1 FC Relevance Matrix

For the FC relevance matrix, we only included results involving connections, as determined by the second condition in Figure 2. For each significant result, we added +1 or −1 to the corresponding FC relevance matrix entry according to the effect direction, that is, +1 if the connection between two regions was positively associated with AUD or AUD severity or −1 if a negative association was reported in the original study. Values were assigned only once per every analysis as it could occur that the same connection would appear more than once in the same analysis, for example with the regions swapped. To enhance the visualization of region‐ and network‐level effects, we used the FC relevance matrix to create connectograms. These connectograms illustrate the connectivity between the regions identified as most relevant in the region ranking and display the proportion of connections positively and negatively associated with AUD relative to all possible within‐ and between‐network connections.

#### 2.4.2 Region Ranking

For the region ranking, we included results involving both connections and single ROIs or networks. In the case of networks, all regions constituting the network were updated by adding +1 to their count whenever the network was reported as significant independent of the direction of the effect. To ensure fairness, each region was updated maximally once per analysis, regardless of how many times it appeared within the same analysis. This approach prevents the overrepresentation of a region that appears in multiple results within the same analysis, such as a region showing decreased connectivity strength across an entire network, from distorting the frequency data. To calculate a region's appearance frequency, we divided its final count by the total number of analyses. For example, a region appearing in significant results in 10 analyses out of 31 would get a score of around 32%.

Furthermore, to better understand the bias generated by seed‐based analyses, we repeated this process generating additional outcomes based exclusively on seed‐based analyses.

## 3 Results

### 3.1 Overview of the Papers Studied

**FIGURE 3:**
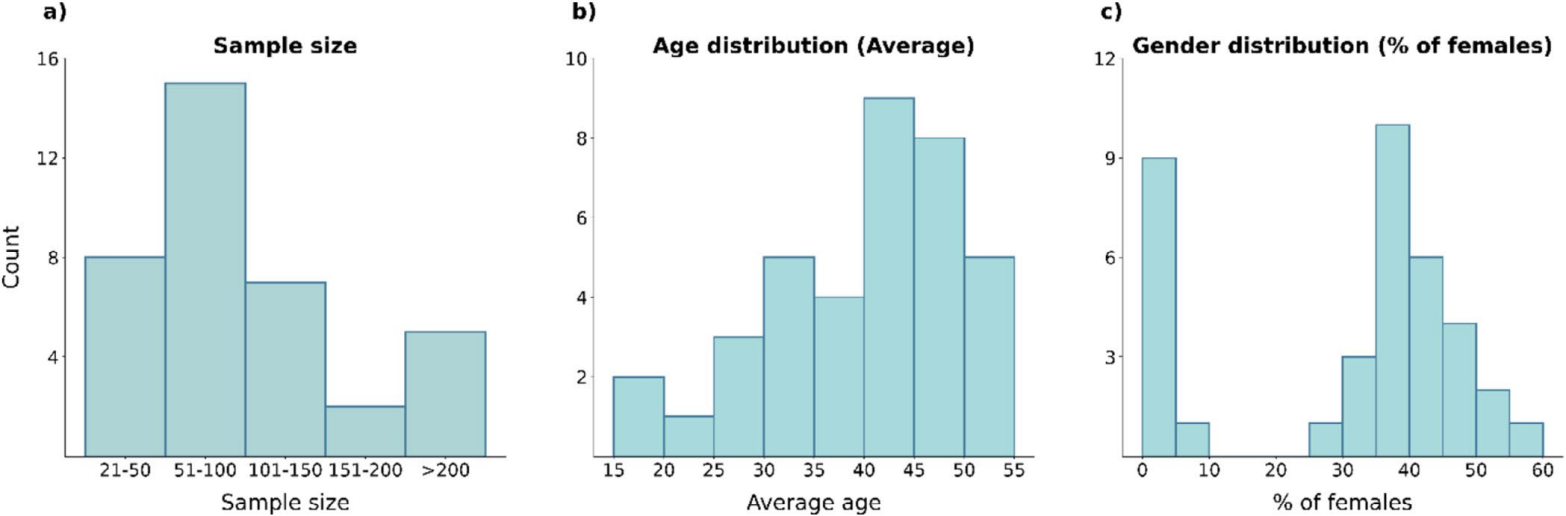
Overview of metadata for all 37 datasets. (a) The distribution of sample sizes. (b) The distribution of average ages. (c) The distribution of gender in the datasets.

The distribution of the sample sizes of the datasets used by the original studies can be seen in Figure 3a. Over half of the studies include less than 100 subjects, with only five datasets from four studies (Gazula et al. 2023; Honnorat et al. 2022; Weiland et al. 2014; Zhu et al. 2022) comprising more than 200 subjects. As shown in Figure 3b, the average age of the participants in the datasets ranges mostly between 40 and 50 years. Figure 3c shows a predominant male representation, with 10 studies (~26%) exclusively using male datasets (Bordier et al. 2022; Deng et al. 2022; Duan et al. 2022; Kamarajan et al. 2022; Kamarajan et al. 2020; Kim et al. 2017; Song et al. 2021; Wang, Fan, et al. 2018; Wang, Zhao, et al. 2018; Yang et al. 2021).

Table 3 provides an overview of the studies' most commonly used analytical methods. A majority use a group comparison approach, with only three studies (~8%) not using group divisions (Dean et al. 2020; Fede et al. 2019; Le et al. 2021). These studies either focus exclusively on AUD subjects or have more than 20 subjects defined as AUD. Among the group divisions, the most common approach is comparing a group of HC participants with an AUD group identified by different diagnostic criteria (~69%), followed by studies comparing relapsers and abstainers (~15%), which usually consist of longitudinal settings to examine relapse factors.

**TABLE 3:** Overview of the methods of interest used by the studies reviewed.

Methods	Papers
**a. Group division**	
AUD versus healthy controls	1, 2, 4, 6, 11, 12, 13, 14, 15, 16, 17, 18, 19, 20, 22, 23, 24, 25, 26, 27, 28, 29, 30, 31, 32, 34, 35, 36, 37, 38
Abstainers versus Relapsers	3, 6, 10, 20, 33, 35
Other	7, 8, 25, 39
No group division	5, 9, 21
**b. Diagnostic criteria used**	
DSM‐IV/DSM‐IV‐TR	1, 6, 10, 13, 14, 15, 16, 17, 18, 19, 20, 22, 25, 26, 27, 30, 33, 35, 36, 37, 38, 39
DSM‐V	2, 7, 11, 28, 32
Recruited from addiction treatment program	3, 5, 8, 9, 29, 31, 36
Other	12, 21, 34
**c. FC inputs used**	
AAL atlas	6, 27, 32
Harvard‐Oxford atlas	3, 5, 20, 25
Other atlases	1, 2, 3, 12, 13, 15, 21, 27, 29, 34, 39
Spherical seeds or masks from the literature	10, 17, 18, 19, 24, 33
Voxel level analysis	7, 14, 35
ICA components	4, 8, 9, 11, 16, 20, 22, 23, 28, 30, 31, 36, 37, 38
**d. Seeds most commonly investigated**	
Nucleus accumbens	2, 10, 13, 20, 21, 35
Anterior cingulate cortex	10, 15, 26
Caudate	3, 13, 20
Orbitofrontal cortex	2, 4, 15
Putamen	3, 13, 20
Reward network	16, 18, 20
Executive control network	16, 20, 34
**e. Outcomes of interest**	
Disorder severity scales: AUDIT	5, 8, 9, 16, 21, 25, 30, 34, 35
Disorder severity scales: ADS	7, 14, 36, 37
raving measures	2, 13, 20, 26
Relapse measures	3, 13, 23, 35
Consumption measures	11, 15, 23
**f. Mediators investigated**	
Structural imaging	2, 4, 24, 27, 29, 33
Impulsivity	17, 18, 25, 33, 37
Genetics	8, 36, 39

*Note:* Each category of methods reports only entries that appeared in at least three different studies, and each entry is linked to the corresponding paper index in Table 1.

Abbreviations: AAL—automated anatomical labelling; ADS—alcohol dependence scale; AUD—alcohol use disorder; AUDIT—alcohol use disorder identification test; DSM—diagnostic and statistical manual; FC—functional connectivity; ICA—independent component analysis.

The primary diagnostic tool used to distinguish an AUD group from HC is the structured clinical interview (SCID) from the different DSM editions, with a prevalence of DSM‐IV/DSM‐IV‐TR (~56%), here aggregated into one group since they don't differ in the diagnostic methods. In other cases, the AUD subjects were recruited through an addiction treatment program.

In the models, the most common FC inputs were atlas‐based parcellations and data‐driven ICA components. The most frequently used atlases were Harvard‐Oxford (Desikan et al. 2006; Frazier et al. 2005; Fritz, Klawonn, and Zahr 2022; Goldstein et al. 2007), appearing in four studies (~10%), and AAL (Tzourio‐Mazoyer et al. 2002) used in three studies (~8%). The ICA approach was used in ~38% of the studies, and the average number of relevant components used, excluding noise, was around 50.

In the 42 seed‐based analyses conducted, over 30 different seeds between ROIs and networks were employed, and Table 3 reports those used at least three times. These include regions linked with AUD in prior research, particularly the Nacc, which featured in six different studies (~15%). Concerning outcomes of interest, the AUDIT score was the most frequently assessed (~23%), followed by the ADS (~10%). Since no other outcome was studied three or more times, the remaining scores are categorized based on aspects of the disorder, such as craving, relapse, and consumption patterns. The most commonly investigated mediators were the structural organization of the brain (~15%), the subjects' impulsivity scores (~15%), and genetic factors (~8%).

### 3.2 FC Relevance Matrix and Most Relevant Connections

**FIGURE 4:**
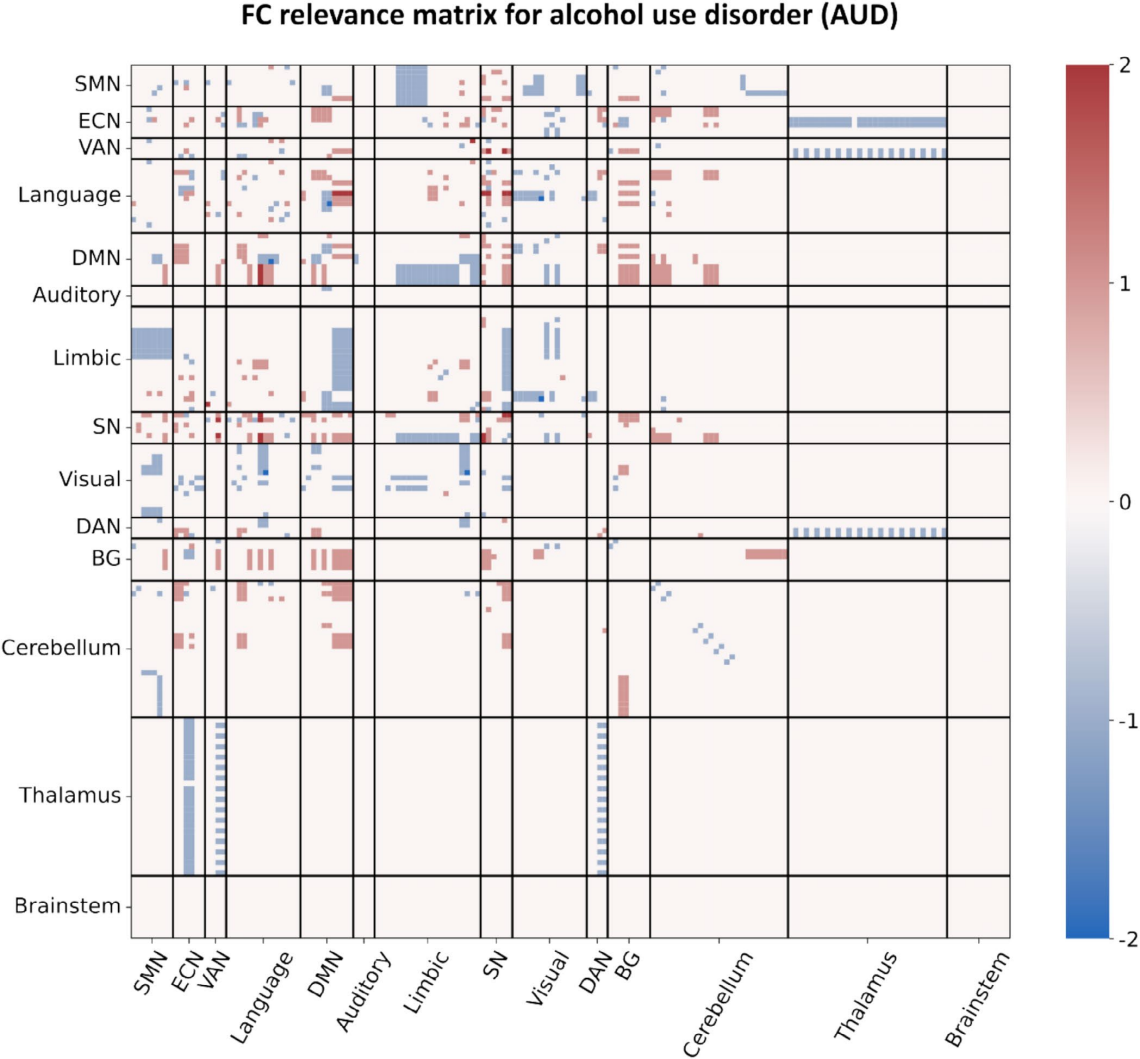
FC relevance matrix showing associations of between‐region connections with AUD, sorted and labeled according to the most common functional networks. AUD—alcohol use disorder; BG—basal ganglia; DAN—dorsal attention network; DMN—default mode network; ECN—executive control network; FC—functional connectivity; L—left, R—right; SMN—sensorimotor network; SN—salience network; VAN—ventral attention network.

Figure 4 displays the FC relevance matrix for connections, with scores ranging from −2 to 2. Of these, 94.3% have a zero score, 2.4% show a positive association with AUD, and 3.3% a negative one. The score is evaluated as the difference between positive and negative findings for each connection. However, only 0.2% of the connections had contrasting results, with 28 connections with a score of zero having one positive and one negative finding and three connections with a score of 1 having two positive and one negative finding. Among the connections with a score of 2, indicating an overall positive association with AUD, we can find a triangle of connections between the insula, ACC (especially supracallosal), and left superior temporal gyrus (STG), and the connection between the opercular part of left IFG and the left middle temporal pole. Among the connections with a score of −2, indicating an overall negative association with AUD, we can find the connection between the superior temporal cortex and right lingual gyrus, as well as between the left middle temporal cortex and the precuneus. All the findings reproduced twice, therefore with a score of 2 or −2, come from independent studies. A list of all the connection scores and the number of their positive and negative associations with AUD, separately for whole‐brain and seed‐based analyses, can be found in Table S4, with the relevance matrix shown in Figure 4 and in Table S2.

**FIGURE 5:**
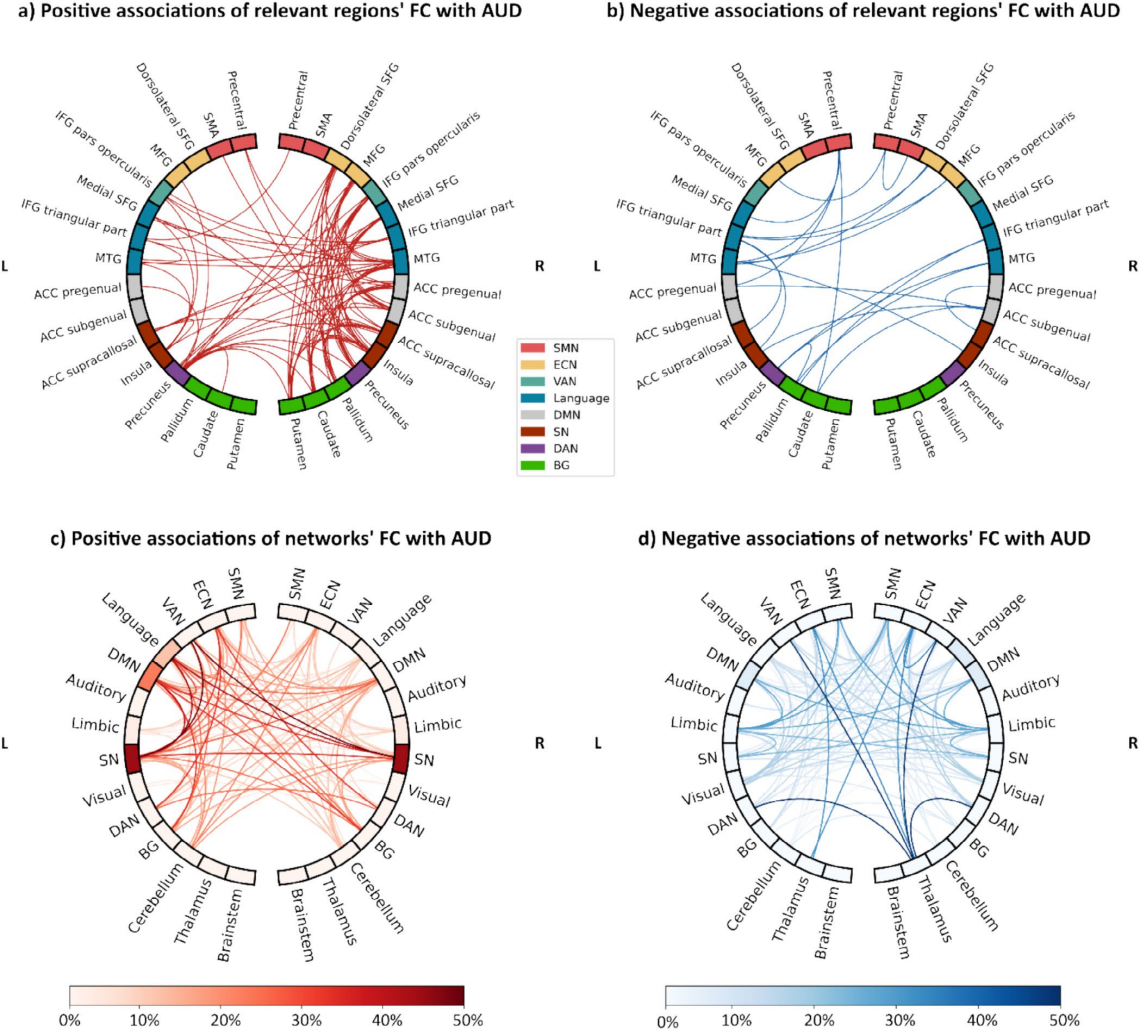
Visualization of FC associations with AUD on both region and network levels. (a and b) Positive and negative associations of FC between the most relevant regions. The nodes are colored according to the network the region belongs to. (c and d) Positive and negative associations of FC at the network level, with the node and edge colors representing the percentage of connections within‐ and between‐networks, respectively, that show significant associations with AUD. ACC—anterior cingulate cortex; AUD—alcohol use disorder; BG—Basal ganglia; DAN—dorsal attention network; DMN—default mode network; ECN—executive control network; FC—functional connectivity; IFG—inferior frontal gyrus; L—left; MFG—middle frontal gyrus; MTG—middle temporal gyrus; R—right; SFG—superior frontal gyrus; SMA—supplementary motor area; SMN—sensorimotor network; SN—salience network; VAN—ventral attention network.

Alternative ways to visualize the connectivity abnormalities shown by the FC relevance matrix are provided by showing the altered connectivity among a selection of the most relevant regions (Figure 5a,b) or aggregating the results by network (Figure 5c,d), or on our online tool ConnExplore. Through this tool, that we developed to visualize connectivity matrices in an anatomical space, the interested reader may select specific ROIs and display the associated connectivity abnormalities on a whole‐brain visualization.

### 3.3 Region Ranking

**FIGURE 6:**
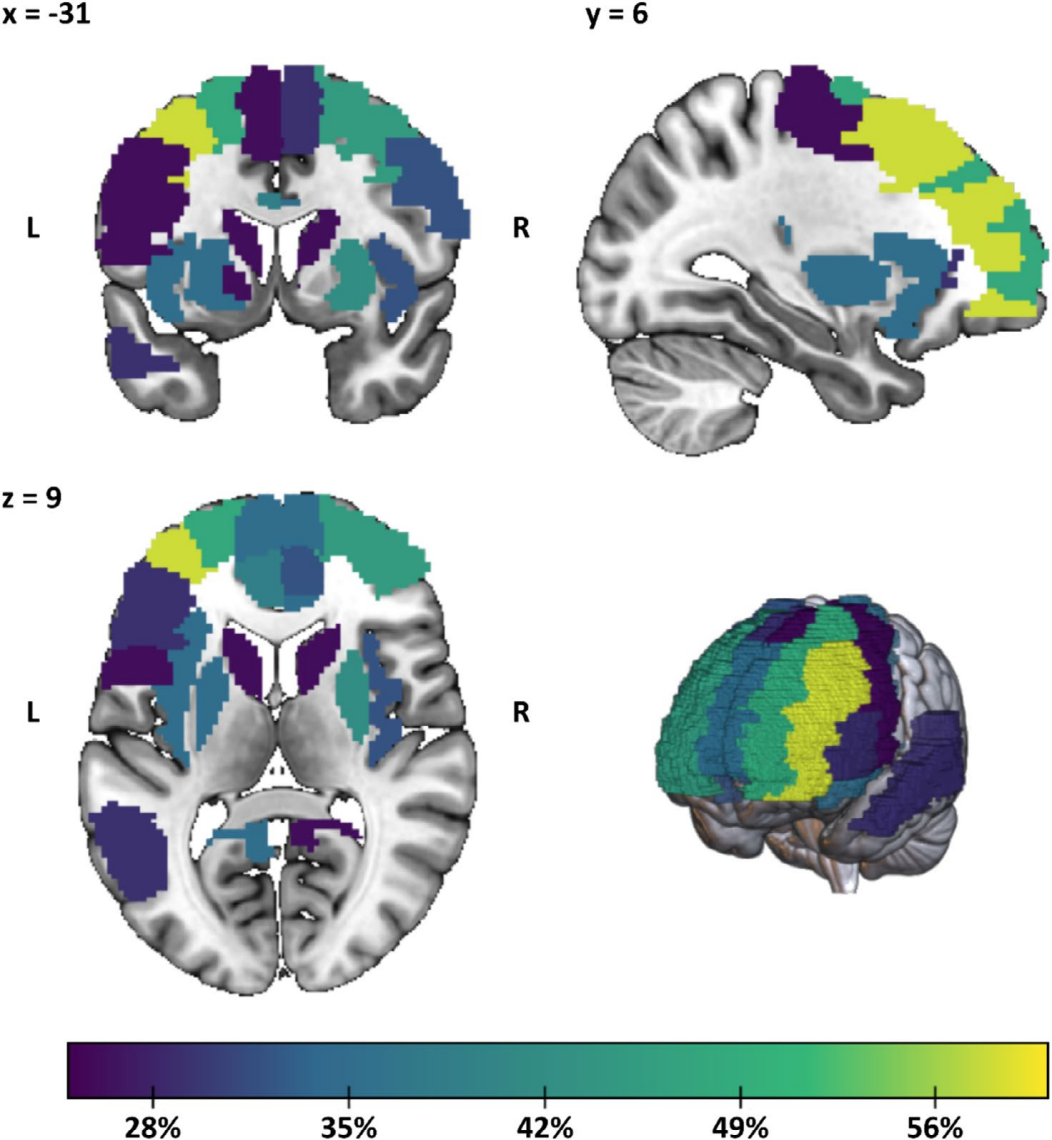
Anatomical visualization of regions identified as most relevant in Table 4. Regions are color‐coded according to the percentage of whole‐brain analyses that reported at least one significant association between AUD and the region's connectivity. Only regions with significant associations reported in more than 25% of the 31 whole‐brain analyses are shown. Coronal (top left), sagittal (top right), axial (bottom left) views, and a 3D rendering (bottom right) are displayed.

Figure 6 and Table 4 show the most critical brain regions according to how often the whole‐brain analyses could find at least one significant association between AUD and their connectivity. Key areas include prefrontal brain regions, such as the MFG and the dorsolateral and medial SFG, along with striatal regions like the putamen, caudate, and pallidum. Other relevant regions include the ACC, insula, and precuneus.

**TABLE 4:** Ranking of the regions of AAL3 associated with significant results in at least 25% of the 31 whole‐brain analyses in at least one of the hemispheres.

Region	LH frequency	RH frequency
Middle frontal gyrus	58%	45%
Dorsolateral superior frontal gyrus	48%	45%
Putamen	35%	42%
ACC supracallosal	39%	33%
ACC pregenual	39%	32%
Medial superior frontal gyrus	35%	35%
Insula	35%	32%
Precuneus	35%	26%
ACC subgenual	32%	32%
Precentral gyrus	26%	32%
Supplementary motor area	26%	29%
Middle temporal gyrus	29%	19%
IFG triangular part	29%	13%
Caudate	26%	26%
Pallidum	26%	23%
IFG opercular part	26%	16%

*Note:* The ranking in the table is based on the highest between left hemisphere (LH) and right hemisphere (RH) frequency for every region.

Abbreviations: AAL3—automated anatomical labelling atlas 3.1; ACC—anterior cingulate cortex; IFG—inferior frontal gyrus.

### 3.4 Comparison With Seed‐Based Region Ranking

A variation of the previous outcome was generated in the context of the seed‐based analyses to show the main biases that their inclusion would have introduced. Table 5a lists the regions most frequently used in seed‐based analyses: some results are similar to Table 4, but the nucleus accumbens and the parietal gyrus are strongly overrepresented. Table 5b sorts their frequencies by their deviation from the whole‐brain region ranking. For instance, the amygdala yielded only one significant result in 31 distinct whole‐brain analyses (Honnorat et al. 2022), but seven among the 42 seed‐based analyses. Conversely, the putamen, the precuneus, SMA, and the supramarginal gyrus, more frequent in whole‐brain analyses, are underrepresented in seed‐based results. The complete region ranking, detailing their frequency across both analysis types, can be found in Table S3. For completeness, we also provide the seed‐based FC relevance matrix in Figure S1 without further discussion.

**TABLE 5:** Region ranking of seed‐based analyses, compared to the one from whole‐brain analyses.

a.				
Region	LH seed‐based	LH Whole‐brain	RH Seed‐based	RH Whole‐brain
ACC supracallosal	43%	39%	43%	35%
Middle frontal gyrus	40%	58%	40%	45%
ACC pregenual	38%	39%	38%	32%
Nucleus accumbens	38%	10%	36%	10%
Dorsolateral superior frontal gyrus	33%	48%	38%	45%
Inferior parietal gyrus	33%	19%	24%	23%
Medial superior frontal gyrus	29%	35%	26%	35%
ACC subgenual	29%	32%	26%	32%
Superior parietal gyrus	26%	13%	26%	6%

b.				
Region	LH seed‐based	LH Whole‐brain	RH Seed‐based	RH Whole‐brain
Nucleus accumbens	38%	10%	36%	10%
Superior parietal gyrus	26%	13%	26%	6%
Inferior parietal gyrus	33%	19%	24%	23%
Amygdala	14%	3%	17%	3%
…	…	…	…	…
Supplementary motor area	5%	26%	7%	29%
Supramarginal gyrus	0%	23%	2%	13%
Putamen	17%	35%	19%	42%
Precuneus	12%	35%	5%	26%

*Note:* (a) Ranking of the regions of AAL3 associated with significant results in at least 25% of the 42 seed‐based analyses in at least one of the hemispheres. The regions are sorted according to the frequency of the highest between their left hemisphere (LH) and right hemisphere (RH) frequency in seed‐based analyses. The second and fourth columns report the respective whole‐brain frequency for comparison; (b) Same frequencies of (a), sorted according to the difference in frequency with the corresponding region in the whole‐brain region ranking.

## 4 Discussion

This systematic review has two main purposes: first, to provide an overview of the literature linking rs‐fMRI to AUD, to gain an understanding of the main methods used in this research area, and second, to provide unbiased quantitative outcomes, namely relevance maps, describing which regions and connections are more often linked to the disorder in the context of rs‐fMRI. Unlike previous reviews discussed in the introduction, our focus is exclusively on the interplay between rs‐fMRI and AUD. We identify key regions recurrently linked to the disorder—either through direct association or via connectivity abnormalities—including the MFG, dorsolateral and medial SFG, ACC, insula, putamen, and precuneus. Given the complexity of interpreting matrices like that shown in Figure 4, we suggest the use of our tool, ConnExplore, for a better visualization of the connectivity abnormalities in an anatomical space.

### 4.1 Overview of Methods and Datasets

The analysis highlights a significant gender bias in existing research, with most of the studies focusing predominantly on male subjects and often excluding females entirely, despite evidence suggesting gender differences in AUD‐related brain (Zhang et al. 2022). This gender discrepancy emphasizes the need for a more inclusive approach in future research, in light of findings that the gender gap in AUD prevalence has been narrowing in recent years (Slade et al. 2016). Furthermore, our examination of the cohorts used across the literature highlights the limited statistical power of most of these studies with more than half including fewer than 100 participants. This could also undermine the reliability of the reported findings and might explain the lack of consistent results in functional connections reported across the 31 whole‐brain analyses, where the highest recurrence of any connection was noted in only two studies. Another explanation for the lack of reliability lies in the heterogeneity of the disorder outcomes examined in the literature (see Table 3a,e), which likely contributes to the variability of the observed associations. However, even limiting our exploration to dichotomous analyses comparing AUD and controls wouldn't give us more control of which aspects are driving the associations between AUD and FC. To address potential biases from individual measures, such as heavy or binge drinking, we ensured that only studies with sufficiently large sample sizes and participants undergoing formal AUD assessments were included.

### 4.2 Relevance Maps

The information on connections and regions more frequently associated with AUD provided by the relevance maps partly aligns with what was previously reported in other reviews (Dupuy and Chanraud 2016; Fritz, Klawonn, and Zahr 2022; Gowin et al. 2023; Nutt et al. 2021): the alterations in the reward system are reflected by disruptions in FC revolving around the SN, with increased within‐network connectivity, especially between the left insula and dACC, and increased between‐network connectivity of these regions with DMN, language network, and dorsal striatum, as shown in Figure 5c. Concerning emotional regulation, the shift in recruitment from temporal limbic regions to prefrontal regions can be seen in the decreased connectivity between DMN, particularly the precuneus, with the left temporal pole, paired with the increased connectivity of DMN regions with the dorsolateral PFC from ECN. Other interesting tendencies are the increased connectivity of the cerebellum mirrored by decreased connectivity of the thalamus, particularly with regions from ECN. Overall, the FC relevance matrix does not agree with a view of AUD as a disconnection syndrome (Dupuy and Chanraud 2016), rather both decreased as well as increased FCs are observed. When looking at Figure 5a,b, which isolates the associations of the connectivity of the most relevant regions, there seem to be more connections strengthened by the disorder as opposed to weakened, especially within the right hemisphere. The region ranking corroborates the relevance of these areas to AUD. The comparison with the seed‐based studies underlines a certain bias in the literature: the most evident case, as shown in Table 5, is that of Nacc, which is chosen as seed in six different papers (Bracht et al. 2021; Fein et al. 2017; Gazula et al. 2023; Kohno et al. 2017; Le et al. 2021; Yang et al. 2021), but only led to three significant results out of 31 whole‐brain analyses, all concerning more general within‐network connectivity of the striatum (Farokhnia et al. 2022; Fede et al. 2019; Zhu, Dutta, et al. 2015). In part, this may be attributed to the relatively small size of the region and weaker signals from this region due to susceptibility dropout and parallel imaging‐related increases in noise (Srirangarajan et al. 2021). Additionally, whole‐brain studies suffer a substantial reduction in power compared to seed‐based studies due to the required application of multiple comparison corrections, a common practice in rs‐fMRI whole‐brain analyses. This phenomenon could also explain the low frequency of other regions relevant to AUD, such as the amygdala and VTA. In other cases, for instance, regarding the parietal gyrus, the cause of the overrepresentation can be indirect, like being connected to a region or belonging to a network, which are often chosen as seeds. The opposite seems to happen with regions like the putamen, SMA, and precuneus. The latter, despite never being chosen a priori as a seed, produced significant results in more than one‐third of the whole‐brain analyses. Together, our observations point to a potential gap in the methodology of existing studies, suggesting a reevaluation of focus areas to enhance the comprehensiveness of alcohol use disorder research.

### 4.3 Limitations

The first limitation of this work is that the body of literature included is relatively small and highly heterogeneous with respect to the methodology, which could explain why most connections only show one significant result or none. This is mainly caused by the field of research being quite new: the oldest among the papers included in this review dates to 2014 and in the excluded ones only 10% date before 2014, with the two oldest papers being published in 2009. In addition, as previously outlined, sample sizes were rather small across studies, thereby contributing to the heterogeneity of findings. These issues also limited our possibilities in applying more refined meta‐analysis approaches to construct the relevance maps. Making every study contribute equally ensures broader inclusion and avoids over‐representation of individual studies in favor of giving more value to a potential replication of a finding. Accounting for study‐specific quality metrics such as sample size, effect size, or methodological robustness would be very challenging and to some degree arbitrary. This limitation stems from inconsistent reporting across studies, which renders the application of a weighting system or further exclusion criteria impractical. In addition, it would further reduce the already limited number of studies included. An example is the consumption of other substances, which might act as confounders: about 60% of the included studies excluded participants with other substance use disorders, some studies don't address the issue, and some just report their presence or treat them as covariates. We therefore decided to only define a minimal sample size as an inclusion criterion (see “2.1. Search strategy and paper selection”). When considering seed‐based analyses, the number of findings to include increases, with a bigger range of scores in the relevance matrix, but they are biased towards the most common seeds and their strongly connected neighbors. Another limitation is the atlas choice: we believe that AAL3 is a good choice for our purpose, but the heterogeneity in the region size can lead to a bias in the regions considered most relevant. For example, the regions composing the frontal lobe are the biggest of the atlas, which could partly explain why some of them are ranked among the most important regions. Moreover, a small number of results involved regions not included in the atlas, and therefore, also not in the relevance maps: Gazula et al. (2023) and Le et al. (2021) found positive associations between AUD and hypothalamus connectivity with middle and superior temporal gyrus and with the hippocampus, and Morris et al. 2016 found negative associations between subthalamic nucleus connectivity and AUD; both regions are not included in the AAL3 atlas.

### 4.4 Future Work

These relevance maps can be used to formulate hypotheses in future projects involving the prediction of AUD from imaging data. For example, multi‐variate machine learning algorithms may generate data‐driven relevance maps based on the FC features importance for prediction, which we would expect to reflect our literature‐driven relevance maps to a certain degree, while potentially incorporating multi‐variate and sometimes non‐linear information that could refine and update the existing relevance maps. Additionally, these maps could be used as a tool to improve seed and feature selection, for example, considering only connections with scores different from zero or only regions with frequency over 25% for prediction purposes. Furthermore, as the field advances and reporting standards improve, future research could explore weighted approaches, potentially enabling the creation of new relevance maps focused on specific aspects of the disorder, such as identifying regions and connections most strongly associated with craving, relapse, or binge drinking, or with subgroups, according to relevant covariates like age and gender. In this way, we would solidify our understanding of the key regions and connections associated with the disorder, and which of these are more useful to characterize it in the framework of resting‐state analysis.

## Conflicts of Interest

The authors declare no conflicts of interest.

## Supporting information


**Figure S1.** Functional connectivity (FC) relevance matrix for seed‐based and whole‐brain analyses showing associations of between‐region connections with AUD, sorted and labeled according to the most common functional networks. SMN—sensorimotor network, ECN—executive control network, VAN—ventral attention network, DMN—default mode network, SN—salience network, DAN—dorsal attention network, BG—Basal ganglia.

**Table S1.** Results table: Overview of all the significant results collected from the studies included in the review, used for the generation of the relevance maps.
**Table S2.** FC relevance matrix: Tabular form of the FC relevance matrix, first outcome described in the manuscript.
**Table S3.** Region ranking: Full list of the regions of interest as defined in the Anatomical Automated Labelling atlas, version 3.1 (AAL3, Rolls et al., 2020) and their respective number of citations and frequency of appearance for whole‐brain and seed‐based analyses separately, sorted in descending order according the frequency of appearance in whole‐brain analyses.
**Table S4.** Connection scores: Full list of the pairwise connections in AAL3 and their respective score and count of positive and negative results for whole‐brain and seed‐base analyses separately, sorted in descending order first according to the score in whole‐brain analyses and then to the one in seed‐based analyses.

## Data Availability

The data that support the findings of this study are openly available in OSF at https://osf.io/gs5b4/?view_only=baa982ae6a98408cb071a7674336d9db.
