# Supplementary material for: Relating Functional Connectivity and Alcohol Use Disorder: A Systematic Review and Derivation of Relevance Maps for Regions and Connections
Source: Hum Brain Mapp. 2025 Feb 7;46(2):e70156. doi: 10.1002/hbm.70156 (PMC11803412; doi:10.1002/hbm.70156)
Supplement: Supplementary file 1 — Figure S1. Functional connectivity (FC) relevance matrix for seed‐based and whole‐brain analyses showing associations of between‐region connections with AUD, sorted and labeled according to the most common functional networks. SMN—sensorimotor network, ECN—executive control network, VAN—ventral attention network, DMN—default mode network, SN—salience network, DAN—dorsal attention network, BG—Basal ganglia. [file HBM-46-e70156-s001.docx]

# Title

**Supplementary materials for**

“Relating functional connectivity and alcohol use disorder: A systematic review and derivation of relevance maps for regions and connections”

# Journal

Human Brain Mapping (https://onlinelibrary.wiley.com/journal/10970193)

# Authors

Marco Bottino^1^, Natálie Bocková^1^, Nico W. Poller^1^, Michael N. Smolka^1^, Justin Böhmer^2,3^, Henrik Walter^2*^, Michael Marxen^1*^

^1^Department of Psychiatry and Psychotherapy, Technische Universität Dresden, Dresden

^2^Department of Psychiatry and Psychotherapy CCM, Charité – Universitätsmedizin Berlin, Corporate Member of Freie Universität Berlin, Humboldt-Universität zu Berlin, and Berlin Institute of Health, 10117 Berlin, Germany

^3^Institute of Medical Psychology, Charité – Universitätsmedizin Berlin, Corporate Member of Freie Universität Berlin, Humboldt-Universität zu Berlin, and Berlin Institute of Health, 10117 Berlin, Germany

*shared senior authorship

# Supplementary figures in this file

- Figure S1

# Other supplementary materials for this manuscript

The supplementary tables cited in the manuscript are in a separate Excel file, one per worksheet

- Table S1 – Results table: Overview of all the significant results collected from the studies included in the review, used for the generation of the relevance maps.
- Table S2 – FC relevance matrix: Tabular form of the FC relevance matrix, first outcome described in the manuscript.
- Table S3 – Region ranking: Full list of the regions of interest as defined in the Anatomical Automated Labelling atlas, version 3.1 (AAL3, Rolls et al., 2020) and their respective number of citations and frequency of appearance for whole-brain and seed-based analyses separately, sorted in descending order according the frequency of appearance in whole-brain analyses.
- Table S4 – Connection scores: Full list of the pairwise connections in AAL3 and their respective score and count of positive and negative results for whole-brain and seed-base analyses separately, sorted in descending order first according to the score in whole-brain analyses and then to the one in seed-based analyses.

# Supplementary figures


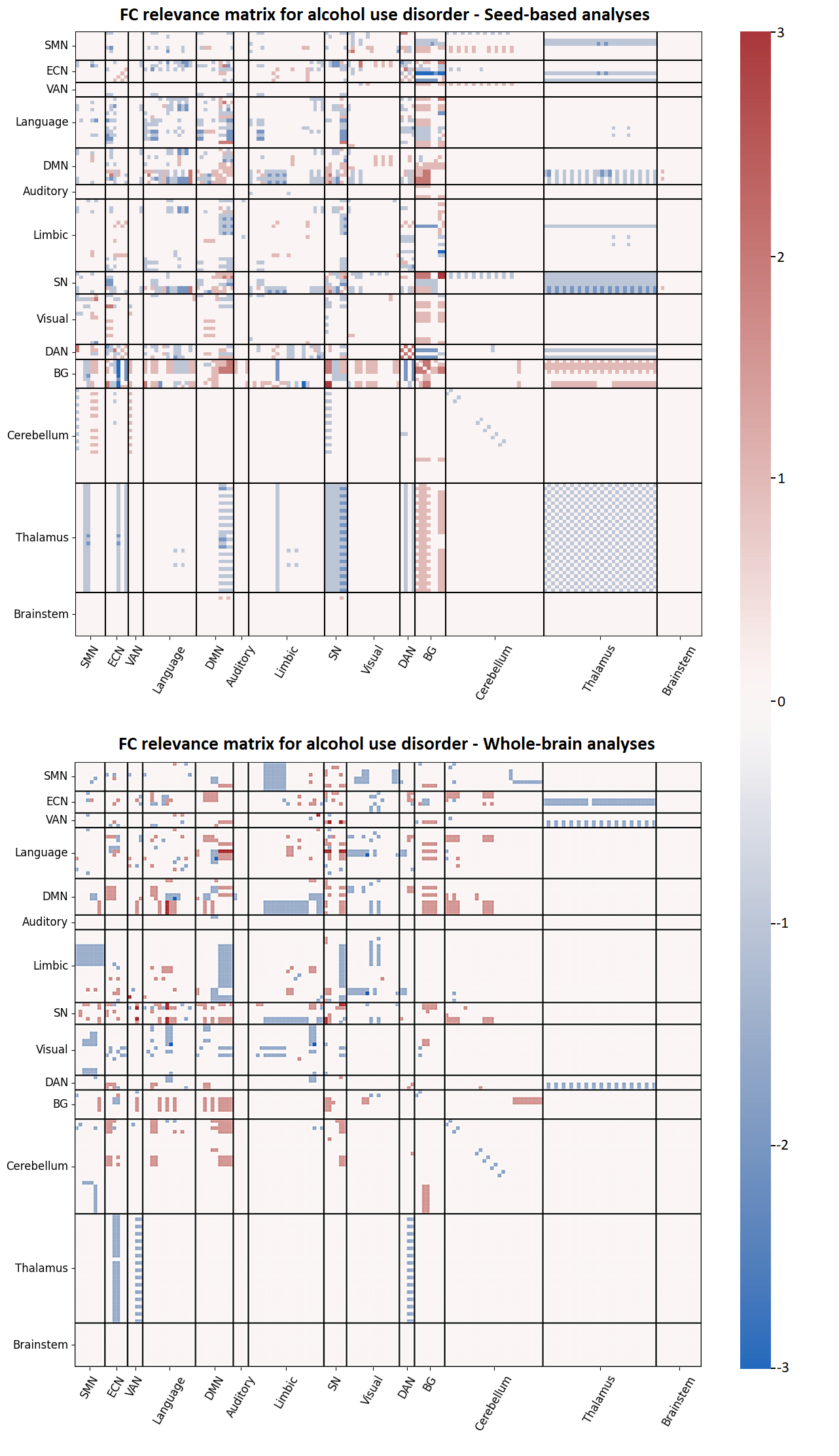


Figure S1: Functional connectivity (FC) relevance matrix for seed-based and whole-brain analyses showing associations of between-region connections with AUD, sorted and labeled according to the most common functional networks. SMN - sensorimotor network, ECN - executive control network, VAN - ventral attention network, DMN - default mode network, SN - salience network, DAN - dorsal attention network, BG - Basal ganglia.

References

Rolls, E. T., Huang, C.‑C., Lin, C.‑P., Feng, J., & Joliot, M. (2020). Automated anatomical labelling atlas 3. *NeuroImage*, *206*, 116189. https://doi.org/10.1016/j.neuroimage.2019.116189
